# Angiotensin-Converting Enzyme 2-Based Biosensing Modalities and Devices for Coronavirus Detection

**DOI:** 10.3390/bios12110984

**Published:** 2022-11-07

**Authors:** Ijaz Gul, Shiyao Zhai, Xiaoyun Zhong, Qun Chen, Xi Yuan, Zhicheng Du, Zhenglin Chen, Muhammad Akmal Raheem, Lin Deng, Edwin Leeansyah, Canyang Zhang, Dongmei Yu, Peiwu Qin

**Affiliations:** 1 Institute of Biopharmaceutical and Health Engineering, Tsinghua Shenzhen International Graduate School, Tsinghua University, Shenzhen 518055, China; 2 Tsinghua-Berkeley Shenzhen Institute, Tsinghua Shenzhen International Graduate School, Tsinghua University, Shenzhen 518055, China; 3 Shenzhen Bay Laboratory, Shenzhen 518132, China; 4 Department of Computer Science and Technology, School of Mechanical, Electrical & Information Engineering, Shandong University, Weihai 264209, China

**Keywords:** COVID-19, ACE2 biosensors, SARS-CoV-2, electrochemical detection, colorimetric sensors, low-cost diagnostic

## Abstract

Rapid and cost-effective diagnostic tests for severe acute respiratory syndrome coronavirus 2 (SARS-CoV-2) are a critical and valuable weapon for the coronavirus disease 2019 (COVID-19) pandemic response. SARS-CoV-2 invasion is primarily mediated by human angiotensin-converting enzyme 2 (hACE2). Recent developments in ACE2-based SARS-CoV-2 detection modalities accentuate the potential of this natural host-virus interaction for developing point-of-care (POC) COVID-19 diagnostic systems. Although research on harnessing ACE2 for SARS-CoV-2 detection is in its infancy, some interesting biosensing devices have been developed, showing the commercial viability of this intriguing new approach. The exquisite performance of the reported ACE2-based COVID-19 biosensors provides opportunities for researchers to develop rapid detection tools suitable for virus detection at points of entry, workplaces, or congregate scenarios in order to effectively implement pandemic control and management plans. However, to be considered as an emerging approach, the rationale for ACE2-based biosensing needs to be critically and comprehensively surveyed and discussed. Herein, we review the recent status of ACE2-based detection methods, the signal transduction principles in ACE2 biosensors and the development trend in the future. We discuss the challenges to development of ACE2-biosensors and delineate prospects for their use, along with recommended solutions and suggestions.

## 1. Introduction

Coronavirus disease 2019 (COVID-19) pandemic is still ongoing, posing a severe threat to human health and global healthcare system. The development of on-demand, rapid and cost-efficient severe acute respiratory syndrome coronavirus 2 (SARS-CoV-2) detection systems is critical to control the unabated pandemic. Although many COVID-19 sensing systems have been developed so far [1], each approach has its pros and cons. Point-of-care testing (POCT) devices are of paramount importance for both resource rich and constrained settings [2]. Introducing a biosensing approach that meets the “ASSURED (affordable, sensitive, specific, user-friendly, rapid and robust, equipment-free and deliverable to end users)” criteria defined by the world health organization (WHO) [3] always remains a prime focus of a biosensing study. Rapid and low-cost diagnostic tests could revolutionize the infectious disease diagnostics by enabling the real-time pathogen detection at population level and assist the government in making timely decisions to curb disease propagation [4]. Although many COVID-9 sensing tools are commercially available, the commercialized methods have various limitations, such as low sensitivity (antigen- and antibody-based testing) and prolonged assay time (nucleic acid testing) [5]. Therefore, novel detection approaches intended to meet ASSURED criteria for SARS-CoV-2 are need of the time.

Based on bioanalyte type, SARS-CoV-2 diagnostics can be categorized into three modalities: (i) nucleic acid detection, (ii) serology testing, and (iii) imaging-based detection (computed tomography) [1]. Polymerase chain reaction (PCR) is a gold standard for COVID-19 diagnosis [6]. Several PCR-based methods have been reported for COVID-19 diagnosis [6]. The limit of detection (LOD) of most of the PCR-based assays is about ≤ 100 copies per milliliter [7], that corresponds to a viral load equal to 2–3 days before the onset of disease symptoms [8]. Among PCR variants, the RT-PCR is a widely used platform for SARS-CoV-2 detection. More than 350 SARS-CoV-2 RT-PCR kits are commercially available [9]. Garg et al. [9] compared the performance of seven commercial RT-PCR tests, including Black Bio’s TRUPCR SARS-CoV-2, Seegene’s Allplex 2019-nCOV test, Lab Gun COVID-19 RT-PCR kit from Lab Genomics, Korea, My Lab’s Patho detect COVID-19 kit, TaqPath RT-PCR COVID-19 kit from Thermo Fisher Scientific, Fosun RT-PCR kit (Fosun Ltd.), and BGI’s real-time RT-PCR kit. The analysis showed that the performance (sensitivity, specificity, and positive and negative predictive values) of BGI, Black Bio, Thermo Fisher Scientific, and Seegene are 100.0%. For weakly positive samples, the diagnostic efficacy of Lab Gun, Fosun, and My Lab is relatively lower. For a detailed account of the performance of SARS-CoV-2 sensing systems, readers are referred to a recent review dedicated to comparative analysis of SARS-CoV-2 diagnostics [7]. However, PCR-based sensing is costly, labor-intensive, and demands experienced users. To overcome these limitations, efforts have been made to develop amplification-free on-demand detection systems [10,11,12]. Unfortunately, the majority of the amplification-free SARS-CoV-2 detection approaches are unable to achieve clinically useful sensitivity for real-sample analysis and continue to recommend nucleic acid pre-amplification prior to testing [13,14], resulting in an increased assay time and cost.

In addition to PCR-based COVID-19 diagnostics, lateral flow assays (LFAs) have shown increased research trend due to certain features, such as rapid response, low-cost, and suitability for both resource-rich and resource-constrained settings. Based on target detection, LFAs are categorized into three types: nucleic acid-, antibodies-, and antigen-based LFAs. According to Global Market Insights (GMI) [15], the LFA market size exceeded USD 7.9 billion in 2021, and a compound annual growth rate (CGAR) of over 5.7% between 2022 and 2028 (>$12.1 billion in 2028) is projected. Commercial LFA products have variable efficiencies depending on the assay conditions. The sensitivities and specificities of the LFAs range from 57.6–100% and 95.87–100%, respectively [16]. A recent study on evaluation of the diagnostic performance of two commercial unsupervised rapid antigen tests reports the sensitivities of 65.7% and 62.1%, while the specificities are 100% [17]. In addition to the signal transduction system used in a detection method, the biocomponent plays a pivotal role in accuracy of the proposed or developed approach. In this regard, SARS-CoV-2 entry receptor Human angiotensin-converting enzyme 2 (hACE2) has enticed research attention as a potential biocomponent.

hACE2 is a well-studied drug target for SARS-CoV-2 treatment [18,19,20]. In early COVID-19 controlling strategies, many studies suggested the possibility of repurposing the drugs effective for other SARS coronaviruses to treat SARS-CoV-2, which could decrease the cost and development time of SARS-CoV-2 therapeutics. The spike proteins of SARS-CoV-1 and SARS-CoV-2 have a sequence similarity of 76% [21], and receptor-binding domains of SARS-CoV-1 and SARS-CoV-2 have an overall sequence similarity of 73–76% [22]. Despite this similarity, a little efficacy against SARS-CoV-2 has been demonstrated in hospitalized COVID-19 cases for SARS-CoV-1 drugs [23]. Several therapeutic approaches targeting ACE2 against COVID-19 have been reported [24], and the virus’s entry into host cells is studied using atomic force microscopy (AFM) [25].

In addition to a potential drug target, ACE2 can be harnessed for the biosensing of SARS-CoV-2. In this regard, recently, ACE2 has enticed substantial research attention as a potential bioreceptor for SARS-CoV-2 detection. Many promising results have been reported where ACE2-SARS-CoV-2 spike protein interaction is translated into a sensitive biosensing signal, leading to a novel COVID-19 sensing modality. Since the ACE2-spike interaction has been thoroughly studied, and virus internalization mediated by ACE2 is a well-established mechanism, repurposing ACE2 as a biosensing modality eliminates the screening time of antibody and aptamers, which might be the most convenient bioreceptor for protein-based biosensing in the near future.

**Figure 1:**
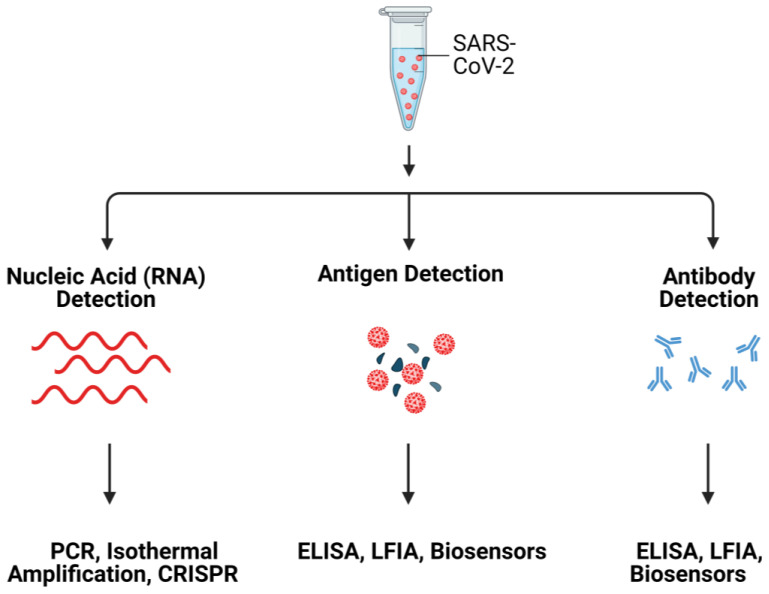
Schematic illustration of SARS-CoV-2 detection systems. Created with Biorender.

Herein, we review the rationale for hACE2 as a potential bioreceptor for detection of SARS-CoV-2. The review starts with a brief introduction to ACE2, followed by a detailed discussion on SARS-CoV-2 detection using ACE2, challenges to ACE2-based detection approaches and possible solutions. Commonly used SARS-CoV-2 detection methods are summarized in Figure 1.

## 2. ACE2: A Mediator for SARS-CoV-2 Invasion into Host Cells

ACE2 is a carboxypeptidase that hydrolyses a single amino acid from the C-terminus of the substrate. ACE2 is the homolog of ACE with 40% amino acid sequence identity. However, the substrate specificity and cleavage pattern of ACE2 are different from those of ACE, a dipeptidase [26]. ACE2 has multiple physiological functions including, catalytic activities with peptide substrates, receptor for SARS-CoVs [27], and amino acid transporter [28]. ACE2 is involved in peptidergic renin-angiotensin system (RAS) that functions in the homeostatic control of cardiovascular and renal systems as well as regulation of extracellular fluid volume [29]. In RAS, the ACE2 converts angiotensin I (ang I) into angiotensin-(1–9) and angiotensin II (ang II) into angiotensin-(1–7) [28,30], where ACE2 plays important roles in the onset and development of myocardial infarction(MI) [29], diabetic cardiovascular complications, systemic and pulmonary hypertension, and COVID-19 pathogenesis [30].

**Figure 2:**
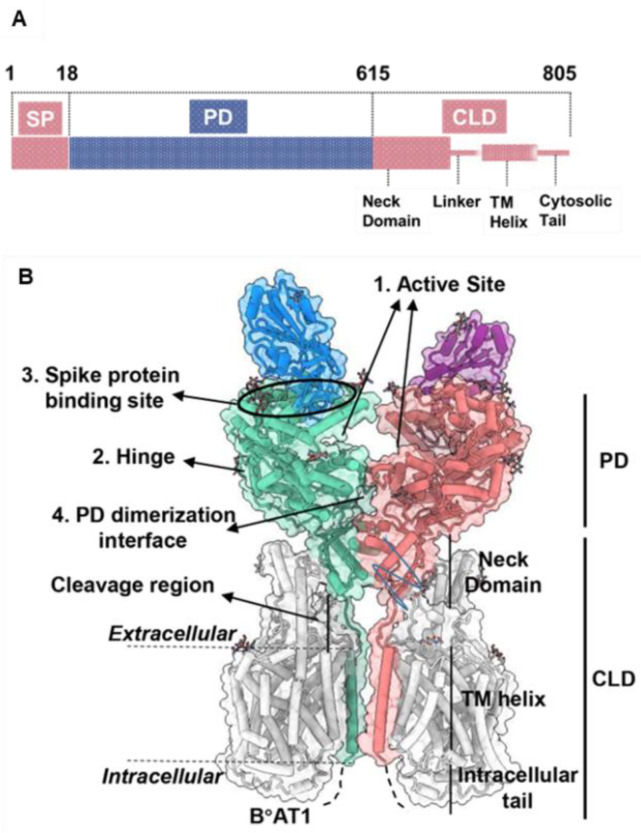
ACE2 gene domain organization and protein structure. (**A**), Full-length ACE2 containing a signal peptide (SP), an N-terminal peptidase domain (PD), and a C-terminal collectrin-like domain (CLD). (**B**), Protein structure of full-length ACE2 dimer in complex with B°AT1 (PDB ID: PDB 6M17). B°AT1 is presented in grey and four key sites (1–4) are highlighted. The numbers (1–805) indicate the amino acid residues. Adapted from Ref. [31] with permission from the publisher. ^©^ 2020 Wiley-VCH GmbH.

The open reading frame of human ACE2 encodes an 805-residue polypeptide. The full-length ACE2 has two domains: a protease catalytic domain (PD) comprising of 598 residues and a collectrin-like domain (CLD) with 190 residues [31]. The PD is present at the N-terminus where a 17-residue signal peptide (SP) is also located and is removed during post-translational modifications. The key sites present in PD include an active site (for peptidase activity), a spike-RBD binding site, a hinge region back of the active site, and a PD dimerization interface (Figure 2) [31]. The hinge region is involved in the movement of the active site, leading to an “open,” “close,” or “intermediate” conformations of the PD. This indicates that the active site and hinge pocket are allosterically linked. Further, spike protein binding to ACE2 increases catalytic activity of the enzyme, which supports the notion that the RBD binding site of ACE2 might be regulated allosterically [32,33]. The CLD is present at the C-terminus and is mainly involved in ACE2 membrane anchorage [31]. The CLD is comprised of a neck domain that is involved in ACE2 dimerization, a cleavage region, a transmembrane helix, and a cytosolic tail.

The full-length ACE2 is a dimer [25]. The dimeric soluble ACE2 (PD domain of the full-length ACE2) shows higher affinity for the S trimer compared to the monomeric soluble ACE2 [34]. In an effort to enhance the S-binding affinity of the soluble ACE2 to use it as a decoy receptor, an ACE2 trimer is created using the foldon tag of bacteriophage T4 fibritin [34]. Due to the fact that all of the ACE2 monomers in the trimeric ACE2 bind to S trimer, the newly engineered ACE2 trimer has a stronger affinity for S trimer. The newly engineered ACE2 trimer showed an enhanced affinity for S trimer due to the involvement of all ACE2 monomers (in the trimeric ACE2) in binding to S trimer.

**Figure 3:**
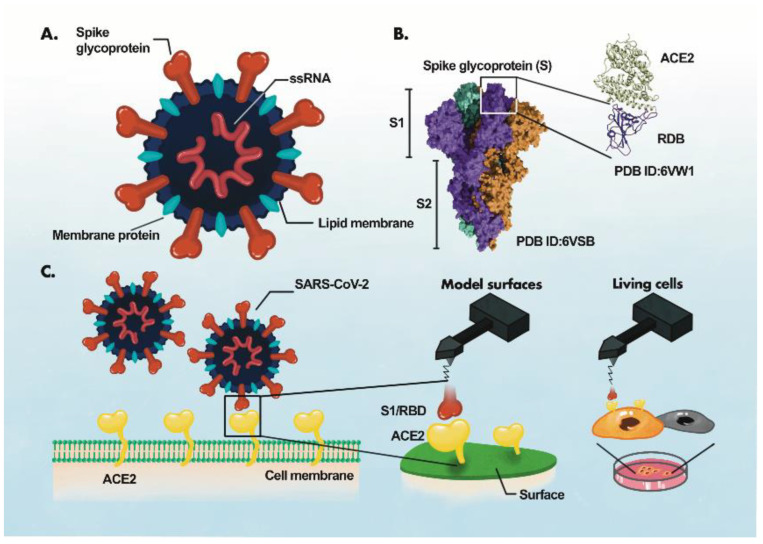
AFM study of ACE2-S- protein binding mechanism. (**A**), Components of the virus particle. (**B**), Complex between receptor-binding domain (RBD) of SARS-CoV-2 and ACE2. (**C**), Atomic force microscopy (AFM) study of the ACE2 binding with RBD. The ACE2 receptor is immobilized on a surface and S1 subunit or RBD is attached to the surface of the AFM tip. Adaptede from Ref. [39] with permission from the author. “This article is licensed under a Creative Commons Attribution 4.0 International License”. http://creativecommons.org/licenses/by/4.0/ (accessed on 25 October 2022).

The identification of the disease-specific biomarkers is the first step towards development of a biosensing system. SARS-CoV-2 is an enveloped, positive-sense single-stranded RNA virus, belonging to the family Coronaviridae [35,36]. SARS-CoV-2 has four structural proteins; spike protein (S), envelope protein (E), membrane protein (M), and the nucleocapsid protein (N)] [36,37], approximately sixteen non-structural proteins and 5–8 accessory proteins [35]. The spike, membrane and envelope proteins (glycoproteins) are present on the outer part of the virus, whereas nucleocapsid protein encapsulates viral genome. The M protein is responsible for virus shape, the E protein is involved in virion assemble and release while the N protein packages the viral genome [38]. The S protein of coronavirus has two functional subunits: S1 and S2. The re ceptor-binding domain (RBD) is present in S1 subunit while S2 subunit mediates the fusion of the host-virus membranes [39]. Schematics of the AFM studies elucidating the molecular mechanism of SARS-CoV-2 binding to the host cell receptor (Figure 3) [39]. The S protein interacts with the ACE2 leading to the virus tight binding with the host cell surface, followed by virus internalization into the host cell. SARS-CoV-2 enters host cells via two distinct pathways, endosomal entry and cell surface entry [40]. In both pathways, the S protein cleavage is a prerequisite for viral entry, and involves two S protein cleavage events. One cleavage occurs at the S1-S2 subunit junction of S protein, while the other occurs at the S2′ site, which lies internal to the S2 subunit. The cleavage of the S1-S2 junction occurs during virus maturation in the infected cells. The cleavage of S2′ leads to the fusion of viral and cellular membranes, resulting in the release of viral RNA into the cytoplasm. Upon binding to ACE2, if the virus-ACE2 complex does not encounter TMPRSS2 or if there is a low expression of TMPRSS2 on the host cell surface, the SARS-CoV-2 is internalized by endocytosis. After endocytosis, the S2′ is cleaved by cathepsins in an acidic environment, which exposes the fusion peptide (FP). In both entry pathways, the S2′ exposes the FP. The conformational changes in S2 resulting from the dissociation of S1 from S2 result in orientational changes in FP, bringing it closer to the target membrane and leading to the release of viral RNA into the host cytoplasm for replication [40]. For further details on mechanism of SARS-CoV-2 entry into host cell, readers are directed to recently published reviews [40,41].

## 3. Harnessing ACE2 as a Bioreceptor

COVID-19 pandemic has attracted researchers from different fields to develop rapid and accurate virus detection systems for mitigation of disease progression in a timely fashion. Despite this surge in COVID-19 biosensing, the developed methods still lack the complete characteristics of an ideal detection system. An ideal biosensing approach should meet the following prerequisites: multiplex detection, high sensitivity for real-sample analysis, high selectivity, rapid response time, portability, disposability, prolonged shelf life, cost-effectiveness, mass manufacturability, and ease of use [38,42,43,44]. Incorporating these features into a new biosensing method or technology always remains the ultimate goal of future detection platforms. In this regard, SARS-CoV-2 biosensors have shown increased research trend since the onset of COVID-19. A biosensor is an integrated device comprising of three components: (i) biorecognition element (bioreceptor); (ii) signal transducer; and (iii) signal detector [45,46]. The interaction between biocomponent and bioanalyte is recognized by the transducer and is translated into a readable signal using digital devices such as embedded devices and smartphones. Biosensors are classified mainly based on the bioreceptor and signal transduction mechanism [46,47]. The use of ACE2 as a bioreceptor for COVID-19 biosensors seems appealing due to certain advantages of ACE2 over other bioreceptors. For instance, a computational analysis shows that the mutations in S-RBD improve the binding of S protein with ACE2, leading to enhanced infectivity [48]. These mutations can lead to false negative results in the case of nucleic acid testing. Since ACE2 is a natural receptor, in contrast to other biorecognition elements, no modifications to ACE2 are needed for variant detection. This ACE2-S protein natural interaction led to the development of ACE2 biosensors for SARS-CoV-2 variants [49,50]. Further, an ACE2 biosensor also detects neutralizing antibodies [50], showing its broad application potential. The amino acid sequence diversity of spike protein among coronaviruses allows for ACE2-S-based SARS-CoV-2 biosensing. The ACE2 can be used as a substitute for the capture antibody to selectively bind the virus, resulting in an ACE2-based immunodetection system where a widely used enzyme linked immunosorbent assay (ELISA) can be easily repurposed into a novel virus assay. Colorimetric detection is a user-friendly approach amenable to automation and realization in POC devices. Similarly, electrochemical detection has garnered significant research attention for the development of POCTs. The binding of ACE2 with the spike protein of the virus may result in an electrochemical event that can sensitively sense the presence of virus [51].

## 4. ACE2-Based State-of-the-Art SARS-CoV-2 Detection

**Figure 4:**
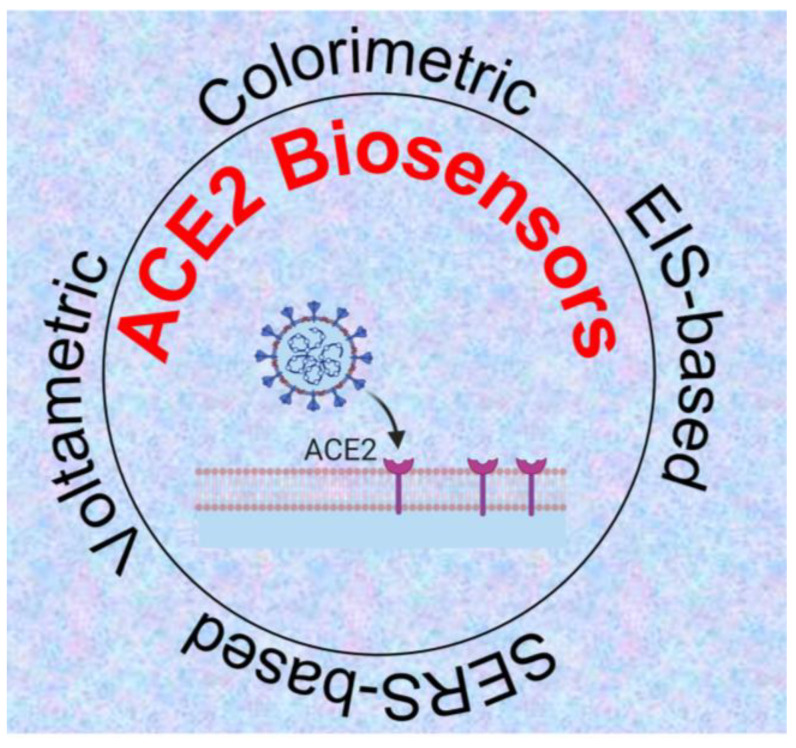
ACE2-based biosensing systems for detection of SARS-CoV-2 [51,52,53,54] Created with Biorender.

The ACE2-virus binding event can be translated into different signal outputs depending on the type of signal transduction system used in the experiment (Figure 4). In the following sub-sections, we discuss these systems in detail.

### 4.1. ACE2-Based Electrochemical Detection of SARS-CoV-2

The electrochemical behavior of the bioreceptor-bioanalyte interaction can be translated into a sensitive electrochemical biosensor. The electrical methods explored for SARS-CoV-2 detection include potentiometry [55], amperometry [56], electrochemical impedance spectroscopy (EIS) [57], differential pulse voltammetry [58,59], and square wave voltammetry [60]. In the amperometric technique, the current generated by the electroactive species is measured and correlated to the analyte concentration [61]. In voltammetry, the potential difference is measured between the working electrode and the reference electrode. The accumulation of a charge potential at the working electrode is measured compared to the reference electrode in an electrochemical cell when zero or no significant current flows between them [58,61]. The bioreceptor-bioanalyte interaction may lead to the change in conductance of the electrolyte solution and can be leveraged as a biosensing signal for developing an impedimetric or conductometric biosensor [62]. In electrochemical biosensors, the electrode is one of the important components. Since the bioelectrochemical event is generally monitored in the close proximity of the working electrode surface, the electrode functionalization with conductive materials and efficient immobilization of the bioreceptor are immensely important to achieve the desirable performance of the biosensor.

**Figure 5:**
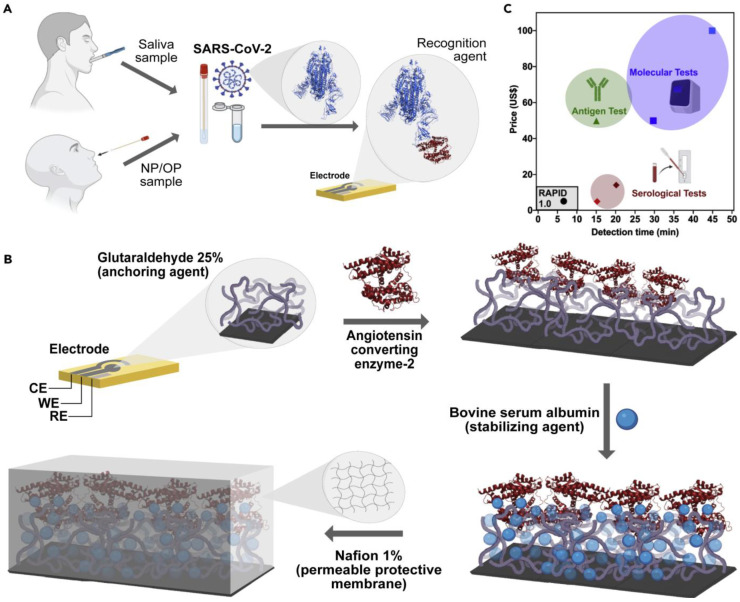
SARS-CoV-2 detection by RAPID test. (**A**), Working principle of RAPID test. (**B**), Functionalization of working electrode. (**C**), Comparison of RAPID test with some FDA-approved tests. Reprinted from Ref. [51] with permission from Elsevier. ^©^ 2021 Elsevier Inc.

**Figure 6:**
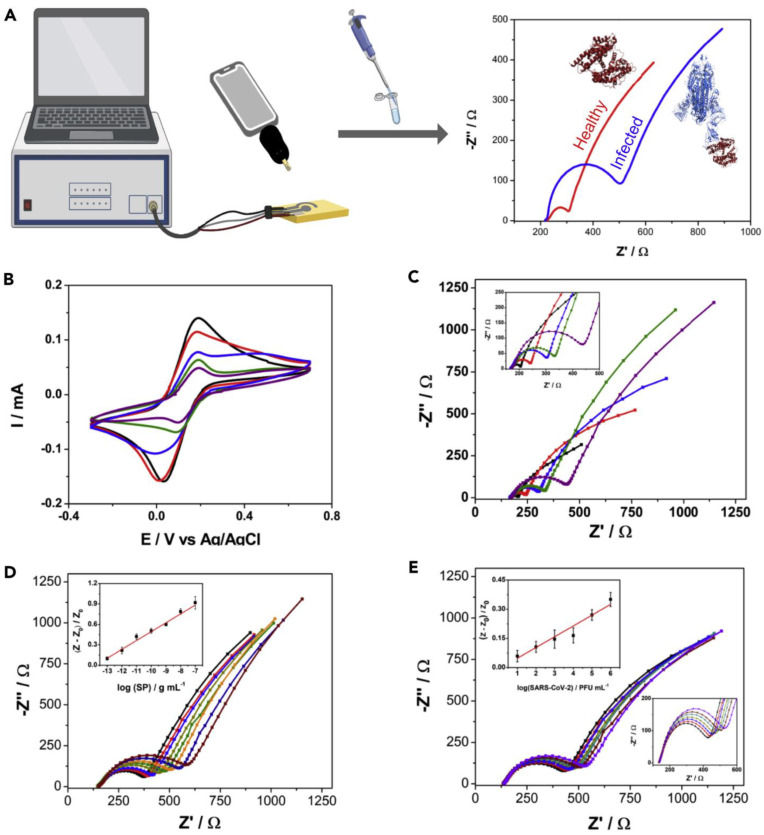
Characterization of the functionalized electrode and development of calibration curve. (**A**), Workflow of the method. (**B**), Cyclic voltammetry (CV) plots of different functionalization steps showing change in current with addition of functionalization layers. (**C**), Nyquist plots of different functionalization steps showing change in charge transfer resistance (R_CT_) with each functionalization step. (**D**), Nyquist plots for varying concentration of spike protein in saliva of a healthy donor. While E shows sensor response to varying concentrations of inactivated virus. Insets in D and (**E**) show linear range of the system. Responses after functionalization with glutaraldehyde, ACE2, BSA, and Nafion are shown in red, blue, green, and purple, respectively, while response of bare electrode is shown in black. Reprinted from Ref. [51] with permission from Elsevier. ^©^ 2021 Elsevier Inc.

An electrochemical SARS-CoV-2 biosensor is developed by Torres et al. [51]. The working electrode is functionalized by the drop-casting method. The ACE2 immobilization on the working electrode is achieved by glutaraldehyde cross-linking. Bovine serum albumin (BSA) is used to avoid non-specific adsorption on the electrode surface (Figure 5). Electrode functionalization is systematically optimized and each fictionalization step is evaluated by cyclic voltammetry and EIS. The CV and Nyquist plots showed a successful functionalization of the working electrode (Figure 6). A miniaturized handheld device called RAPID (Real-time Accurate Portable Impedimetric Detection) is developed. The binding of the target analyte with ACE2 causes a change in interfacial electron transfer kinetics between the redox probe in solution and conducting electrode sites. Electrochemical impedance spectroscopy (EIS) is employed to detect the sensing signal. The calibration curves obtained with two different samples (spike protein and inactivated virus) show a satisfactory sensitivity and robustness of the method (Figure 6). The use of Nafion as a protective layer (and high concentration of ACE2) contribute to the sensitivity and robustness of the biosensor. The sensitivity and specificity of the RAPID for nasopharyngeal/oropharyngeal swabs and saliva samples are 85.3% and 100%, and 100% and 86.5% respectively, with an assay volume of 10 µL and a response time of 4 min. The detection limit of the biosensor is 2.18 fg mL^−1^ (in buffer) and 1.39 pg mL^−1^ for saliva samples.

In another development [24], the palladium nano thin film electrode (Pd-NTF) is fabricated and functionalized by exploiting Pd-S bond formation between the active Pd surface and ACE2. 1-octadecanethiol is used as blocking agent. The functionalized electrode is subsequently coupled to the EIS to realize a complete biosensor. The practical utility of the platform is demonstrated by screening modulators of SARS-CoV-2 S-protein-ACE2 interaction using a very small assay volume. By using the developed platform, potential pharmacological therapy that leads to suppressed virus binding with ACE2 is also identified.

**Figure 7:**
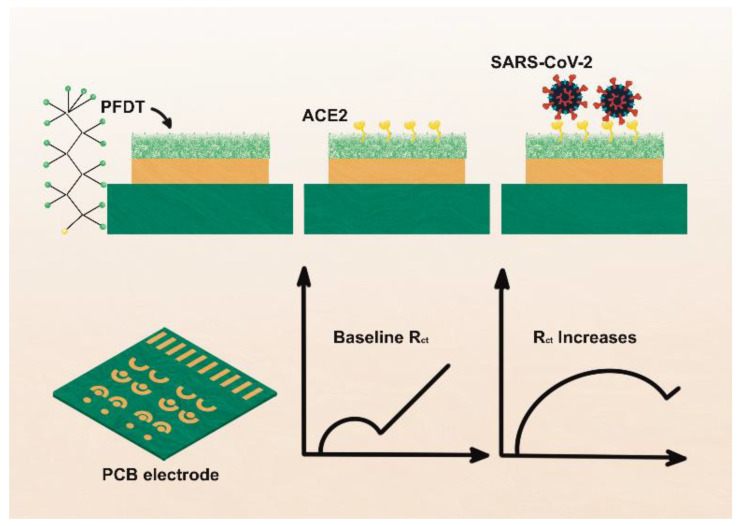
Schematic representation of the fabrication of PFDT-ACE2 biosensor. Top portion shows the sensor fabrication procedure and bottom part shows sensor signals obtained using electrochemical impedance spectroscopy. PFDT, 1H1H,2H,2H-Perfluorodecanethiol; R_ct_: Charge transfer resistance. Adapted from Ref. [65] with permission from the Royal Society of Chemistry. This article is licensed under a Creative Commons Attribution 3.0 Unported Licence. https://creativecommons.org/licenses/by/3.0/ (accessed on 21 October 2022).

ACE2 has two hydrophobic regions, the 18-amino-acid signal peptide on the N-terminus and a 22-amino-acid region on the C-terminus of the enzyme. The hydrophobic region facilitates its anchoring to the cell membrane [63] and allows ACE2 immobilization on amphiphobic structures resembling to cell membrane. Fluorous self-assembled monolayers (SAM) have been used in thin-film transistors (TFTs) to reduce biofouling of material surface [64]. Based on antibiofouling behavior and possibility of effective enzyme immobilization on electrode surface, Vezza et al., [65] deploy a perfluorocarbon self-assembled monolayer of 1H,1H,2H,2H-Perfluorodecanethiol (PFDT) on gold sensor surface to form an amphiphobic surface where ACE2 is immobilized by physisorption on PFDT (Figure 7). The sensor is challenged to the streptavidin, interleukin 6 (IL6), viral transport medium, and saliva samples to test its specificity. The biosensor shows clinically relevant analytical properties.

**Figure 8:**
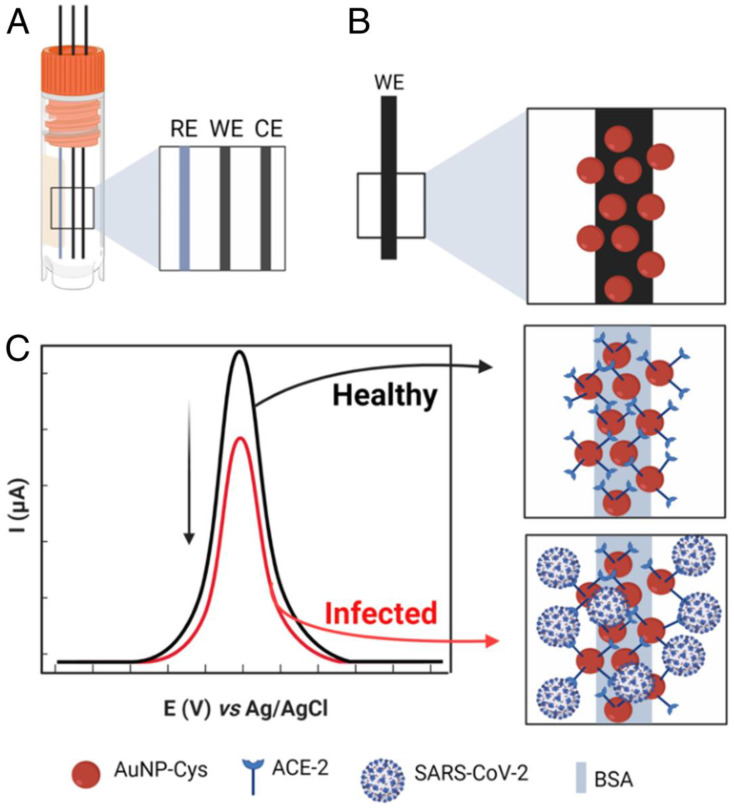
Schematic illustration of the biosensor fabrication and detection principle. (**A**), Electrode system of the developed sensor. (**B**), Functionalization of working electrode. (**C**), Detection principle. RE, reference electrode; WE, working electrode; CE, counter electrode. Adapted from Ref. [52] with permission from authors. Copyright © 2021, the authors. This article is licensed under a Creative Commons Attribution license 4.0 (CC BY) https://creativecommons.org/licenses/by/4.0/ (accessed on 21 October 2022).

A low-cost biosensor, LEAD (Low-cost Electrochemical Advanced Diagnostic) composed of pencil graphite electrodes, is developed by de Lima et al. [52]. The transducer is modified with gold nanoparticles (AuNPs), cysteamine (cys), and bovine serum albumin (BSA) by using different cross-linkers. The ACE2 is anchored on the modified electrode, resulting in the ACE2-AuNPs-cys (Figure 8). The use of gold nanoparticles increases the electrode’s sensitivity due to efficient electron transfer between the electrode and the redox probe ([Fe(CN)_6_]^−3/−4^). The biosensor could detect 229 fg mL^−1^ spike protein. Further, the practical utility of the system is demonstrated by detecting virus in clinical samples. Moreover, the biosensor is insensitive to other potential interfering viruses with shelf-life of 5 days. Rapid response time and the use of easily accessible and commercially available materials are the key attributes of the developed biosensor.

### 4.2. ACE2-Based Optodiagnostics for COVID-19

**Figure 9:**
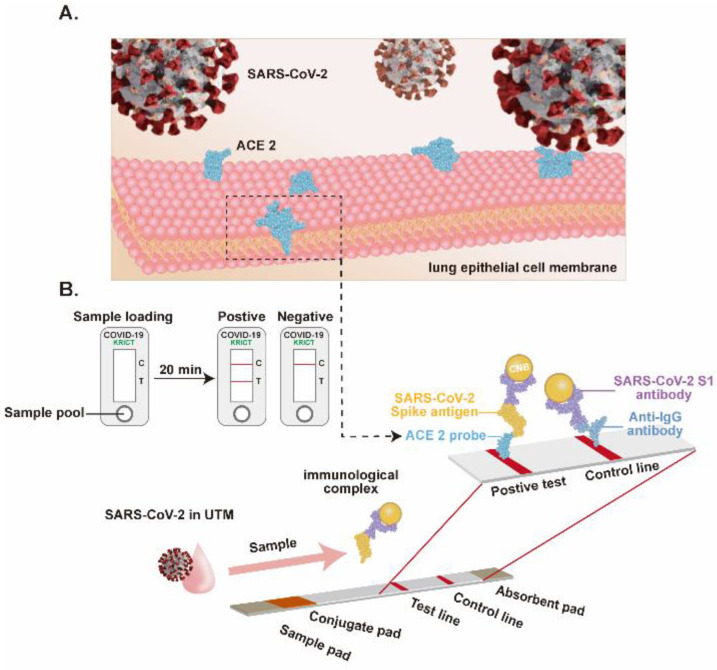
Lateral flow assay-based SARS-CoV-2 detection. (**A**), The cellular receptor of the SARS-CoV-2, the ACE2 expressed on lung epithelial cells. (**B**), Schematic of the LFIA for SARS-CoV-2 detection. Adapted from Ref. [53] with permission from Elsevier. ^©^ 2020 Published by Elsevier B.V.

Immunodetection systems have garnered substantial research attention due to their certain features, such as rapid response, ease of fabrication, and low cost. The use of an appropriate antibody pair for SARS-CoV-2 detection may improve the sensitivity and reliability of the system. In this regard, a rapid SARS-CoV-2 detection method based on ACE2 (Figure 9) is developed [53], where ACE2 could form a matched pair with commercial antibodies. The ACE2 is used as a capture probe to selectively bind the SARS-CoV-2 S1 subunit of the S protein. Three different commercially available antibodies (CR3022, F26G19, and S1-mAb) are systematically evaluated to find the best pair with ACE2. Interestingly, the developed assay is more sensitive to the RBD than the S1, indicating the enhanced affinity of ACE2 for RBD. Spike proteins from other related viruses, such as SARS-CoV-1 and Middle East respiratory syndrome coronavirus (MERS-CoV) show no cross-reactivity. The LOD of the developed system is 1.86 × 10^5^ copies per milliliter, and this is the first study to detect COVID-19 antigen in a lateral flow format using ACE2 as a capture probe. The study opens avenues to develop mutation-free virus detection assays where bioreceptor-virus interaction can be realized as a detection approach. A förster resonance energy transfer (FRET) phenomenon is exploited to develop a SARS-CoV-2-derived RBD ACE2 biosensor [66]. The FRET probe is developed by labelling RBD with YPet and Turquoise2-GL as acceptor and donor, respectively. The biosensor could sensitively detect the interaction between hACE2 and the RBD fragment derived from the SARS-CoV-2 spike protein, demonstrating its potential application in drug design.

In another development [67], a smartphone-assisted ACE2-based optodiagnostic system is designed and developed. Gold nanoparticles and cysteamine are used [52] and fabricated into a user-friendly sensing system. The cotton swab is functionalized with ACE2, and ACE2 is immobilized on the AuNP-cys. The ACE2-functionalized cotton swab is dipped into the sample where the analyte (spike protein or virus particle) is captured by the ACE2. After that, AuNP-cys-ACE2 is added to the reaction system and a color change is observed. The color change is attributed to the binding of virus/spike protein with ACE2, resulting in a sandwich formation that leads to plasmonic change in the AuNPs. The analytical features of the assay are comparable to the other related reports [68,69]. Prolonged incubation (>5 min) of the assay enabled adsorption of the functionalized nanoparticles onto cotton swabs, resulting in a false positive result. The use of blocking agents or a relatively inert support material may decrease the unwanted interactions of the functionalized AuNPs.

### 4.3. SERS-Based Detection

The development timeline of COVID-19 detection technologies indicates that most of the reported methods are particularly focused on SARS-CoV-2 detection in biological fluids. Waste water-based epidemiology (WWE) has been employed to detect different biomarkers, such as pharmaceuticals, drugs, industrial chemicals, and biologicals in wastewater [44]. Human viruses have been detected in wastewater [70,71]. The proper monitoring of viruses in environmental water can help in pandemic preparedness [72]. For example, SARS-CoV-2 RNA was detected in sewage waters in the Netherlands [73] and Australia [70]. The copy number per mL significantly increased with the increasing prevalence of the virus in the population, indicating the potential of sewage water surveillance as a promising tool to monitor the virus load in a population. The discovery of the enhancement of Raman scattering induced by molecules adsorbed on the surface of nanostructured metals has revolutionized the analytical techniques to achieve desired sensitivities. In the surface-enhanced Raman scattering (SERS) phenomenon, the light scattering is greatly enhanced by molecules adsorbed onto the corrugated metallic surfaces, leading to single-molecule SERS sensing [74]. The approach has demonstrated the single-molecule sensitivity [75,76,77].

**Figure 10:**
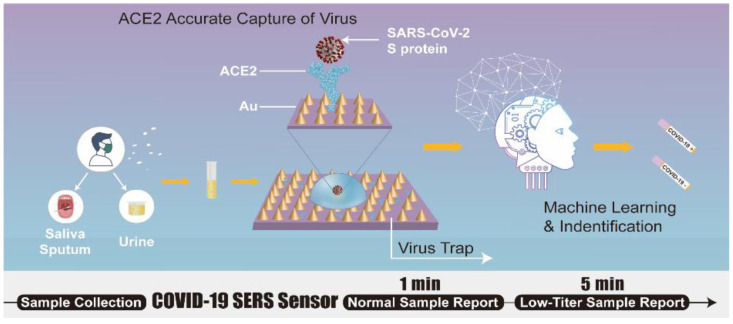
Schematic of SERS biosensor based on ACE2 for SARS-CoV-2 detection. Adapted from Ref. [54] with permission from authors. Copyright © 2021, the authors. This article is licensed under a Creative Commons Attribution 4.0 International License http://creativecommons.org/licenses/by/4.0/ (accessed on 12 October 2022).

In an effort to detect SARS-CoV-2 in environmental samples and harness the latent potential of SERS technology to combat the COVID-19 pandemic, a SERS sensor is designed and developed by Yang et al. [54] (Figure 10). The sensor, hACE2 immobilized gold-nanoneedle arrays (GNAs), shows a 10^6^-fold virus enrichment attributed to the strong ACE2 binding affinity for SARS-CoV-2 and the unique geometry of the GNAs obstructing virus escape. The strong SARS-CoV-2 binding affinity of the ACE2 and the oblique hierarchical structure of the GNAs, and electromagnetic field effect coupling, synergistically result in a 10^9^-fold enhancement of the SERS signal and a detection limit of 80 copies mL^−1^. The biosensor integration with machine-learning methods (principal component analysis and discrimination analysis) reliably differentiates the SARS-CoV-2 SERS signal from that of SARS-CoV-1. The practical application of the system is demonstrated by using urine samples spiked with virus, indicating the potential of SERS sensor for virus detection in wastewater.

## 5. Brief Comparison of ACE2-Based SARS-CoV-2 Diagnostics with Other Sensing Modalities and Sensing Layers

Rapid and accurate diagnostic tests are of paramount importance, not only for screening patients but also for the success of drug and vaccine development programs. Current COVID-19 testing involves two major strategies, viz., detection of virus particles (by virus culture), viral RNA and proteins from oropharyngeal or nasopharyngeal samples, and detecting SARS-CoV-2 antibodies [78]. In addition to these tests, olfactory tests using “electronic nose” [79,80,81,82] and “detective dogs” [83] have also been proposed, but these studies need further validation.

To employ DNA detection platforms for SARS-CoV-2, the virus RNA needs to be reverse transcribed and then PCR-amplified for detection. Many versions of PCR for real-time SARS-CoV-2 detection have been developed by repurposing the technology introduced for SARS-CoV-1 and MERS-CoV [84,85,86] to expedite the COVID-19 diagnosis and mitigation plans. Since PCR-based molecular testing is a laboratory-based time-consuming approach, providing space for research on the development of PCR-independent detection approaches. Towards this end, reverse transcription loop-mediated isothermal amplification (RT-LAMP) methods have shown increased research attention and practical implementation. The approach is independent of the need for the thermocyclers and could be realized for naked eye detection in less than 50 min [87]. Thus, RT-LAMP-based assays may complement RT-PCR in future. Since nucleic acid detection targets the specific regions of the genome, mutations in the target region may affect the accuracy of the assay, leading to false negative results. To overcome these limitations, the optimization of primers/probes has been proposed [88], which may likely reduce the false negative results.

CRISPR-Cas (Clustered Regularly Interspaced Short Palindromic Sequences (CRISPR)-CRISPR-associated proteins (Cas) technology has gained traction for nucleic acid detection. The CRISPR technology has been integrated with isothermal amplification approaches for sensitive detection of virus. For instance, a CRISPR-Cas12-based SARS-CoV-2 detection assay is developed by combining RT-LAMP for RNA amplification and CRISPR-based selective detection in an LFA format [89]. The assay can detect virus in less than 40 min using off-the-shelf reagents and easy-to-use LFA strips. The accuracy of the assay is comparable to that of qPCR. Although promising, the method still relies on RNA extraction and pre-amplification for CIRSPR-based detection. CRISPR-based diagnostics have been optimized and improved for amplification-free [90,91,92] and RNA extraction-free [93] SARS-CoV-2 detection.

Immunodiagnostic platforms have also been developed for SARS-CoV-2 detection. In immunosensing, antigens bind to the host cell receptor, whereas antibodies bind to antigens, resulting in a biological event that is translated into a sensing system [94,95]. These tests can rapidly identify people capable of spreading infection. Ventura et al. [96] developed a colorimetric COVID-19 diagnostic. The gold nanoparticles (AuNPs) are functionalized with antibodies against the E, M, and N proteins of SARS-CoV-2. In the presence of analyte (SARS-CoV-2), the functionalized AuNPs form a layer on the virus surface. The plasmonic effect of AuNPs leads to a color change of the reaction system that is correlated to the presence of the virus. The assay is more than 95% sensitive and specific. Monoclonal and polyclonal antibodies are widely used biocomponent for viral detection [88]. Monoclonal antibodies (mAbs) are more specific than polyclonal antibodies (pAbs), but mAbs are relatively costly due to their prolonged production time [97,98]. On the other hand, antibody single-chain variable fragments (scFvs) have also been employed in virus immunosensing. These scFvs (28 kDa) are stable and show less variability compared to the complete antibody, but purification of single-chain fragments needs additional processing steps [98,99]. Another small-sized (15 kDa) distinct antibody fragment, called “nanobody,” derived from the heavy-chain-only antibodies from sera of camelids [100] has recently shown promising applications in SARS-CoV-2 diagnostics. The small size, stability, ability to detect antigenic sites inaccessible to antibodies due to the larger size of antibodies, and ability to bind the non-epitopic sites of the antigens are the merits of nanobodies [100]. Field effect transistor (FET)- [101] and differential pulse voltammetry (DPV) [102]-based SARS-CoV-2 detection systems have been developed using nanobodies. Satisfactory analytical figures of merit are depicted by these sensors. Although nanobodies possess most of the features of an ideal probe, the small size of the probe necessitates the oriented immobilization on transducer, increasing the cost and labor of the detection system.

Peptides are emerging as a promising sensing layer for COVID-19 diagnostics. ACE2-derived peptides have shown satisfactory results for colorimetric [103] and electrochemical [104] detection of SARS-CoV-2. The small size and commercial viability of the peptides make them an attractive alternative. Further, the peptides which do not compete with ACE2 for binding are suitable as an orthogonal affinity probes for SARS-CoV-2 diagnosis [105]. Although peptides are appealing probes for SARS-CoV-2, the longer (and hydrophobic) peptides are difficult to synthesize. Further, their affinity for S_RBD_ is relatively lower than the ACE2 [103]. The approaches for designing peptides for SARS-CoV-2 are not yet well developed.

Aptamers are single-stranded oligos that can fold into a specific structure to bind targets with high selectivity and affinity. Most of the reported S protein-binding aptamers have dissociation constants (K_ds_) ranging from 2–85 nM [106], which are comparable to antibodies. Some multimeric aptamers targeting S protein have shown pM affinities (2.1–2.3 pM) [106], indicating a significant improvement in binding affinities compared to other sensing layers. Similarly, N protein-binding aptamers have also been reported [107], but S-binding aptamers are widely studied. In addition to improved binding constants, the aptamers are enticing in terms of higher thermal stability, smaller size, and easy and specific modifications [107]. In addition, a recent study reports on aptamers that can selectively bind different SARS-CoV-2 variants [108], paving the way towards the development of universal aptamer probes. Despite the potential advantages mentioned above, the aptasensors are not yet universally adapted. That might be due to the unavailability of a standard protocol for the selection of aptamers for different analytes of interest [109]. Although efforts have been made to improve aptamer stability [110], aptamers are relatively less stable in biological media due to the presence of nucleases [109].

Another sensing approach is the indirect detection of viruses by detecting antibodies produced against invading viruses. These antibody tests may help to determine the infection timeline. The satisfactory sensitivity and specificity have been obtained using an assay for simultaneous detection of immunoglobulin G (IgG) and IgM [111]. Since this assay detects antibodies against infection, it cannot give information about active infection *i.e*., presence of virus in the sample. However, this method is appropriate for deciding a treatment plan. The presence of particular antibody could be used to estimate the stage of infection. For example, detection of IgM suggests an acute infection, whereas the presence of IgG indicates a chronic or previous infection [112].

The low-cost, rapid response time, accuracy, and mass producibility of the reported ACE2-based sensing methods open up avenues to develop novel biosensing devices. If disease prognosis and treatment differ between mutant strains in the future, a biosensing system to detect viral strains would be a valuable COVID-19 sensing tool. In this regard, receptor-virus interaction can be studied to screen variants in a population in a cost-effective manner. The key features of various ACE2-based COVID-19 detection methods are summarized in Table 1, the Table 2 shows a summary of SARS-CoV-2 detection methods, while a direct comparison of ACE2 with other related bioreceptors is presented in Table 3.

## 6. Challenges and Perspectives for ACE2-Based Biosensing Systems

Biosensors are portable, cost-effective, and rapid diagnostic devices with proven clinical applications for some diseases. The glucose biosensor is the best example of a successful biosensor [133]. Although research on diagnostics has already been accelerated in response to COVID-19 pandemic, the development of an accurate, on-demand, rapid, sensitive, selective, mass-manufacturable, and economic COVID-19 detection system is a daunting challenge. However, these features are prerequisites for the COVID-19 sensors for pandemic control. The ACE2-based sensing devices have shown promising results in the lab, but practical implementation of the reported approaches may face certain challenges due to some limitations discussed below.

The ACE2-based sensing devices developed so far meet some of the above-mentioned merits, such as low-cost, rapid response, and mass-manufacturability (due to integration with commercially available technology). The specificities and sensitivities of the reported sensors are mainly studied using simulated samples, so real-sample analysis at a relatively larger scale is not well-demonstrated and may have different results when the sensor is challenged to a larger sample size. Further, the possible interference from other structurally or functionally related components of the biofluid is a common challenge to all sensors, and also implies to ACE2 biosensors. The commonly used strategies to reduce noise include selective separation of the analyte of interest from the complex biological sample and sample dilution using assay buffer prior to detection that may also be adopted for ACE2-based biosensors. A novel truncated ACE2 isoform (delta or dACE2) that is expressed in epithelial cells and upregulated in response to respiratory virus infections and interferons has been reported [134,135]. The reports on dACE2 conclude that the upregulation of dACE2 is unlikely to influence the host susceptibility to SARS-CoV-2 infection as dACE2 lacks spike binding affinity and peptidase activity. Hence, presence of dACE2 should not affect the reliability of ACE2 biosensors. In addition, a study on ACE2 expression dynamics reports a differential expression of ACE2 in the normal and COVID-19-affected human brains [136]. Similarly, a research on ACE2 expression in olfactory epithelial cells in mice models shows an increased expression of ACE2 in the older mice [137]. The presence of ACE2 in real samples might have an impact as a competitor. Therefore, these issues should be taken into account when considering ACE2 biosensors for clinical applications.

The effective immobilization of the bioreceptor on transducer is of utmost importance for selective detection. The ACE2 immobilization on electrode surfaces in the SARS-CoV-2 biosensors is achieved using conventional techniques such as adsorption and carbodiimide chemistry. Traditional protein immobilization methods may result in a blockage of the bioreceptor’s active site or binding site, resulting in decreased or even diminished biorecognition of the analyte. In the case of ACE2 biosensors, this challenge can be addressed by exploiting the potential of protein engineering-based oriented immobilization of the bioreceptor on the support surface [138], that may retain the binding ability of the receptor close to its ability in the solution phase. For instance, the use of SpyTag/SpyCatcher mediated oriented immobilization can be an effective approach. SpyTag/SpyCatcher peptide pair obtained from *Streptococcus pyogenes* [139] can form a spontaneous isopeptide bond within minutes [140]. This pair is approximately 20 times more stable (mechanostability) than the widely employed biotin-streptavidin complex [139,141]. The SpyTag/SpyCatcher pair is used for oriented immobilization of the antibodies that resulted in a 5-fold improvement in the LOD of the system [142]. Similarly, a nanobody-SpyCatcher complex is immobilized on the gate electrode of the field effect transistor (FET) to obtain an orientation-controlled bioconjugation of a self-assembled monolayer (SAM) [101]. The use of linker renders flexibility to the nanobody (bioreceptor). High specificity and single-molecule detection sensitivity are obtained using a very small sample volume of 5 µL and a response time of about 10 min. Similarly, some other immobilization approaches can be explored to improve the analytical characteristics of ACE2 biosensors [143,144].

Since the function of ACE2 in human organs is to cleave peptide substrates such as angiotensin I and II [145], we anticipate that the presence of peptide substrates in the biological samples might have an impact on analytical sensitivity of the system. Further, the presence of ACE2-targeting drugs in real samples may also lead to noise. The selectivity of some reported ACE2 biosensors is tested using viruses and proteins as potential interferents. The effect of the natural substrates of ACE2 on the clinical sensitivity of the system may give insights on the robustness of these biodevices. Another approach to improve ACE2 biosensor selectivity is to explore ACE2 variants as potential bioreceptor. ACE2 polymorphism has been discussed in a review [145]. Some reports have shown that genetic variations in ACE2 and some epigenetic factors play role in individuals’ susceptibility to COVID-19 infection [146]. The engineered ACE2 and ACE2-based peptides can be promising bioreceptors for selective and sensitive detection of SARS-CoV-2. Recently, Zhang et al. reported DNA aptamers with enhanced affinity for S protein [106]. The affinity of the reported aptamers is higher than that of antibody. Since ACE2 is a widely studied receptor, its engineering (like above-mentioned aptamers) for biosensing applications might be an interesting future research.

**Figure 11:**
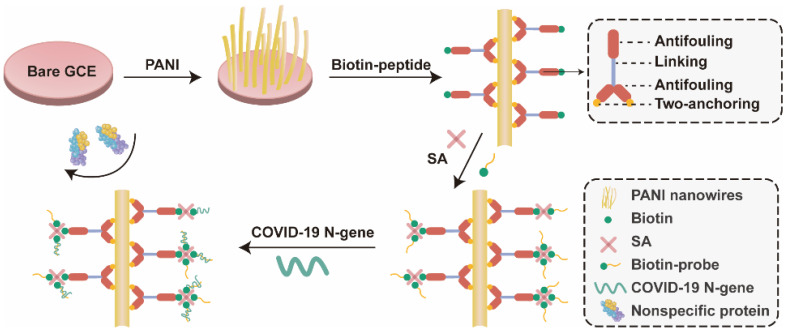
Schematic representation of the antifouling COVID-19 sensor. PANI, polyaniline; SA, streptavidin; GCE, glassy carbon electrode. Adapted from Ref. [155] with permission from the American Chemical Society. ^©^ 2021, American Chemical Society.

The sensitivity of the device is its ability to detect target at a lower concentration in the presence of potential interferents. The spacing between the biocomponent and transducer, and selective capture of the target by a bioreceptor are important players that influence the overall sensitivity of the biosensing system, depending on the sample preparation and biofunctionalization approaches used for sensing. If specificity of the sensing layer is assured then lower detection limits could be achieved. In addition, the sensitivity of the transducer is equally important to achieve desired detection limits. In ACE2-based biosensing, the integration of highly sensitive nanoarchitectures with existing approaches may further improve the sensitivity of the sensing devices [147]. Further, the use of microfluidics for sample pretreatment and its integration with ACE2 biosensing might also be well-placed to achieve lower detection limits [148,149]. The incredible potential of electrochemical biosensors for real-sample analysis has been well-demonstrated by glucose sensor(s) [150]. Despite successful application of glucose biosensor, the use of electrochemical biosensors for other biomarkers is not yet well-explored. The electrode fouling caused by non-specific binding of biomolecules on interface is the major obstacle in harnessing the potential of electrochemical biosensors, and deleteriously affects the sensitivity of the biosensor [151,152,153,154]. The use of semi-permeable membrane to permeate glucose (a small molecule) and filter-out the larger molecules helped to overcome this limitation for glucose biosensor [150,154], resulting in a sensitive detection. Introducing a novel antifouling system for ACE2-based electrochemical biosensor could improve the sensitivity of the biosensor. For instance, Song et al. [155] designed a Y-shaped peptide as an antifouling agent and developed a highly sensitive genosensor based on electrochemical signal transduction to detect N gene of the SARS-CoV-2. The electrode is functionalized with polyaniline (PANI) and newly designed peptide is anchored on the PANI-functionalized electrode. The two chains of the Y-shaped peptide are anchored on the PANI and cover maximum interface (hence, reduce the non-specific binding). The biotin-streptavidin interaction is leveraged to immobilize the probe on the main chain of the Y-shaped antifouling peptide (Figure 11).

Poor storage stability of the ACE2 biosensors may affect their practical implementation in pandemic control and management. The medical devices having long shelf-life can easily be made available to public for self-testing. In this way the disease may be curtailed at the point-of-infection/source. Therefore, further efforts should be devoted to improve the operational stability and shelf-life of the ACE2 biosensors by exploring advanced immobilization approaches such as metal organic frameworks [156] and other simple and effective immobilization approaches [157]. For instance, the protein (enzyme) stabilization by single enzyme nanocapsules (SENs) has enticed substantial research attention. In this approach, the protein (especially enzyme) is encapsulated in a thin polymer, prepared either by in situ polymerization or wrapping a preformed, permeable, and thin polymer around it [158,159,160]. For example, glucose oxidase (GOX) nanocapsules are prepared by a two-tier polymerization approach to obtain an active and stable biocatalyst. For this, enzyme surface modification is done by vinyl/acryloyl groups by acryloxylation. After that, the acryloxylated enzyme is encapsulated by a thin permeable nanoshell, that renders stability to the immobilized biocatalyst [149,161]. Additionally, the application of protein engineering approach for discovering highly stable ACE2 variants might be a good future research.

**Figure 12:**
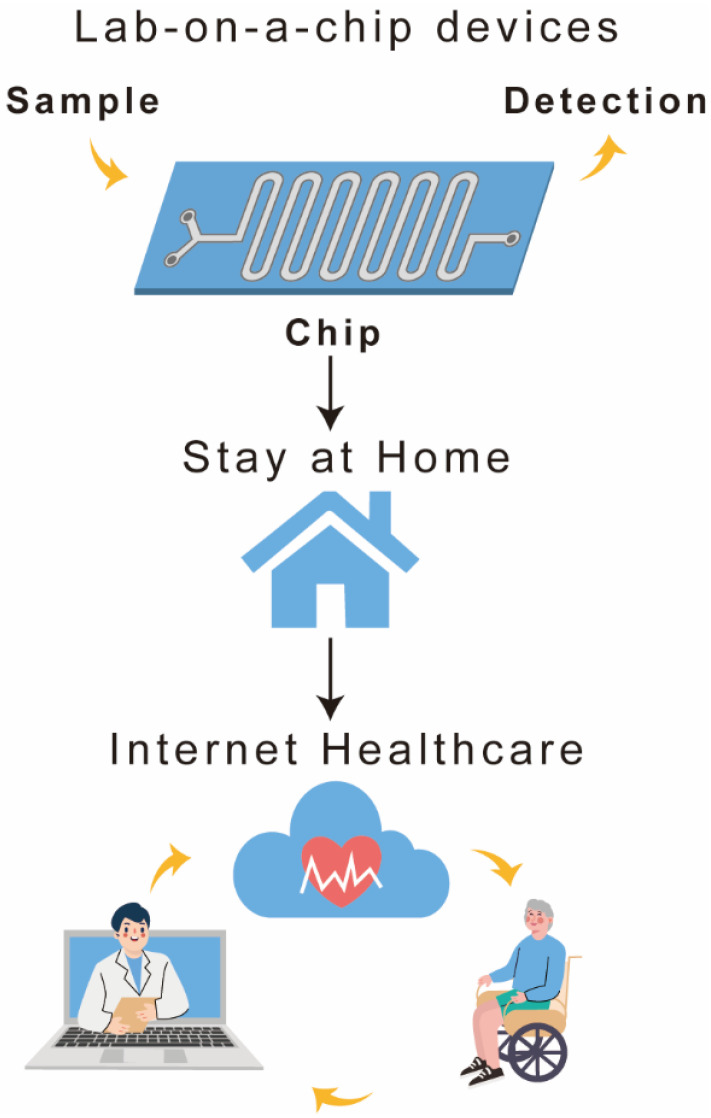
Schematic representation of the IoMT system.

The integration of portable devices with a biosensing set-up is a step forward towards digital technology for healthcare. The reported ACE2 biosensors have been combined with smartphones to realize instrument-free signal detection. Efforts are being made to develop an internet of medical things (IoMT)-based disease diagnosis, treatment, and surveillance system. In IoMT, the biosensing device is wirelessly connected to a portable data acquisition and processing device, connecting patients, health experts, and healthcare centers [162] (Figure 12). The IoMT approach has shown significant growth over the past few years [163,164] and has potential to contribute in fight against ongoing COVID-19 pandemic.

## 7. Conclusions

Diagnostic tools for SARS-CoV-2 have rapidly increased since its first report in 2019. ACE2-based COVID-19 sensing systems have recently garnered increased research attention due to their appealing features, viz., rapid response, high sensitivity, portability, user-friendliness, miniaturization, and amenability to automation. Since ACE2 biosensors mimic host-virus natural interaction, the sensing approach might be considered a ‘mutation-free’ detection modality. The application of smartphone as a detector has been nicely demonstrated in the reported ACE2-based devices, paving the way for digital healthcare technology. Based on signal transduction mechanism, ACE2 biosensors are classified into electrochemical, colorimetric, and SER biosensors. Compared to other sensing modalities, ACE2-based electrochemical approach is relatively well-explored. Among electrochemical biosensors, the EIS remained an obvious choice for biosensor development. Although FETs have shown promising results in biosensor development, the ACE2-based FET biosensors are not yet well-explored, providing space to develop FET-ACE2 biosensors for enhanced sensitivity. Most of the reported sensors achieve satisfactory detection limits in spiked samples. However, there needs to be an extensive clinical validation of the devices. Compared to other SARS-CoV-2 tests, research on ACE2-based COVID-19 diagnosis remains in infancy, necessitating additional concerted research endeavors to unleash the biosensing potential of ACE2.

Even though the ACE2-based sensing approach has fascinated progress, there are certain problems associated to these sensing methods that may hinder their practical application. Matrix effect, electrode fouling, mass transport issues, and complex sensor fabrication procedures are common problems. In the case of ACE2, no satisfactory storage stability is observed in almost all reported ACE2 biosensors. This problem needs to be addressed by optimizing bioreceptor immobilization conditions, reagents used for the immobilization process, developing novel fabrication approaches, and improving stability of the transducer. Since ACE2-RBD interaction is an important drug target, the presence of drugs may interfere with the biosensor signal. Although it is a common problem associated with antigen tests, the robustness of the ACE2 biosensors should be thoroughly investigated and effects of vaccines should also be considered. For this, multiplex detection system can be developed to assure the reliability of the test. Further research is needed for rigorous clinical validation of the ACE2 biosensors. It is anticipated that the ACE2-based sensing modalities can be instrumental in developing point-of-care sensors to meet ASSURED criteria, and could be helpful in combating COVID-19 pandemic [165,166]. The detection approaches discussed in this manuscript have potential to be extended for diagnosis of other viruses such as the recently re-emerged monkeypox virus [167] and amphibian viruses [168].

## Figures and Tables

**Table 1:** Summary of ACE2-based SARS-CoV-2 biosensors.

Bioreceptor	Target	Detection Method	Immobilization/Fabrication	Support Material	Detection Limit	Shelf-Life (Room Temperature or 4–8 °C)/Portability	Time	Clinical Samples Used and Sample Size	Ref.
ACE2	S protein	EIS	ACE2 anchoring on working electrode by GA	Carbon electrode	2.18 fg mL^−1^ S protein	7 days/portable	4 min	Yes (*n* = 151)	[51]
ACE2	S protein	EIS	ACE2 physiosorption on PFDT-functionalized working electrode	Gold surface	1.68 ng fg mL S protein	Shelf-life not reported/portable	30 min	Yes (*n* = 2)	[65]
ACE2	S protein	SWV	ACE2 immobilization on working electrode by EDC-NHS	Graphite electrode functionalized with AuNPs-cys	229 fg mL^−1^ S protein	5 days/portable	6.5 min	Yes (*n* = 113)	[52]
ACE2	S protein	Chronoamperometry	A sandwich ELISA-type immunoassay	Gold electrode	22.5 ng mL^−1^	Shelf-life not reported/portable	91.5 min	Yes (*n* = 4)	[113]
ACE2	S protein	Colorimetric	ACE2 immobilization by EDC/NHS	Cotton swab and gold nanoparticles	0.154 pg mL^−1^	7 days/portable	5 min	Yes (*n* = 100)	[67]
ACE2	S protein	SER	ACE2 adsorption on amide-modified gold nanoarray	Gold nanoarrays on silicon substrate	80 copies mL^−1^	Shelf-life not reported/portable	5 min	Yes (sample size not mentioned)	[54]
ACE2	S protein	LFA	ACE2 adsorbed on nitrocellulose membrane	Nitrocellulose membrane	1.86 × 10^5^ copies mL^−1^ in clinical samples	Shelf-life not reported/portable	20 min	Yes (*n* = 4)	[53]

**Table 2:** List of some recently reported sensing systems for SARS-CoV-2.

Category	Bioreceptor	Target	Detection Method	Immobilization/Fabrication	Support Material	Detection Limit	Time	Ref.
	CRISPR/ Cas12a	N-gene	Colorimetric	Liquid-phase detection using AuNP-labeled ssDNA	N/A	1 copy µL^−1^	90 min	[13]
	CRISPR/ Cas13a	N-gene RNA	Fluorescence	Liquid-phase detection in microchamber array device	N/A	10 fM	<5 min	[91]
CRISPR-based	CRISPR/ Cas12a	N-gene RNA	Nanopore sensor	Liquid-phase detection	N/A	22.5 aM	>30 min	[114]
	CRISPR/ Cas12a	RdRp gene (RNA dependent RNA polymerase)	Electrochemiluminescence (ECL)	DNA tetrahedron immobilized on functionalized glassy carbon electrode for ECL signal	N/A	23.7 aM	>60 min	[115]
	CRISPR/ Cas12a	Open reading frame 1ab (ORF1ab) and N-gene	LFA	Analyte-biocomponent reaction in liquid-phase	N/A	7 copies per reaction	~60 min	[116]
	CRISPR/ Cas12b	Open reading frame 1ab (ORF1a/b) and N-gene	LFA	Analyte-biocomponent reaction in liquid-phase	N/A	10 copies per reaction	60 min	[117]
	CRISPR/ Cas12b	ORF1ab, N- and O- gene	Colorimetric (magnetic pull-down)	Analyte-biocomponent reaction in liquid-phase	N/A	50 copies per reaction	>50 min	[118]
	Antibody for N protein	N protein	SWV	N protein immobilized on cotton-tipped SPE	nanofiber-modified SPE	0.8 pg mL^−1^	> 20 min	[119]
	Antibody for S protein	S protein	FET sensor	Antibody for S protein immobilized on the graphene sheets of FET (immobilization on channel)	Graphene with Au/Cr electrode layer	1 fg mL^−1^	/	[120]
	Antibody for S protein	S protein	Chronoamperometry	Antibody immobilization by conjugation with DNA liker with a tethered ferrocene probe	Gold coated chip	1 pg mL^−1^	5 min	[121]
Immunosensors	Antibody for S protein	S protein	DPV	Antibody immobilized by EDC/NHS on cysteamine-modified Au electrode	Gold-modified laser scribed graphene	2.9 ng mL^−1^	~4 min	[122]
	Antibody for N protein	N protein	Amperometry	Capture antibody immobilized on screen-printed gold electrode, while sensing probe prepared by dually labeling magnetic bead with HRP and anti N protein antibody	screen-printed gold electrode and magnetic beads	10 pg mL^−1^ (in diluted serum)	<1 h	[123]
	Antibody for N protein	N protein	Chemiluminescence	Antibody immobilized on magnetic beads functionalized with -AuNP and modified with Co^2+^	Gold nanoparticle-functionalized magnetic beads	69 fg mL^−1^	20 min	[124]
	Antibody for N protein	N protein	Chemiluminescence	Antibody immobilized on AuNP attached to N-(4-aminobutyl)-N-ethylisoluminol and Co^2+^-functionalized MBs	Functionalized magnetic beads	21 fg mL^−1^	25 min	[125]
	DNA aptamer	S protein	EIS	Aptamer immobilized on functionalized gold electrode	Gold electrode	1000 viral particles per mL (in saliva sample)	<10 min	[106]
	DNA aptamer	S protein	Photoelectrochemical sensing	DNA aptamer immobilized on photoactive material (Au NPs/Yb-TCPP) coated on a glass carbon electrode	Au NPs/Yb-TCPP composite [Ytterbium Tetrakis(4-carboxyphenyl) porphyrin (Yb-TCPP)]	72 ng mL^−1^	>70 min	[126]
Aptasensors	DNA aptamer	S protein	Proximity ligation assay	Liquid-phase detection coupled with qPCR	N/A	37.5 pg mL^−1^	~2 h.	[127]
	DNA aptamer	S protein	Surface plasmon resonance (SPR)	Aptamer immobilized on polyethylene glycol interfaced gold nano-film deposited on optical fiber	Gold nano-film immobilized on optical fiber	36.7 nM	~10 min	[128]
	DNA aptamer	S1 subunit of S protein	Localized Surface Plasmon Resonance	Biotinylated aptamer immobilized on nano-gold surface by biotin-streptavidin interaction	Gold surface	0.26 nM	~16 min	[129]

**Table 3:** Comparison between ACE2 and other related bioreceptors.

Bioreceptor	Binding Affinity (Kd nM) *	Reference	Specificity	Ease of Synthesis	Development Stage	Effect of Mutation on Assay Performance
ACE2	0.06–319.7	[34,53,130,131]	High (so far)	Easy	Laboratory technology	Less **
Antibodies	0.001–185.1	[53,132]	High (may vary in case of antibody escape)	Time-consuming	Commercialized	More ***
Peptides	14.5–31.9	[103]	Satisfactory	Easy but may vary based on peptide length and other properties	Laboratory technology	Might be more ****
Nanobodies	5–37	[101,102]	Satisfactory	Time-consuming	Laboratory technology	Might be more ****
Aptamers	0.0021–85	[106]	Satisfactory	Initial screening is time-consuming. Once available, chemical synthesis of shorter oligos is easy.	Laboratory technology	Might be more ****

* Binding affinities for ACE2 are obtained from the publications focused on therapeutic applications of ACE2. Please note that the binding affinities of the above-mentioned bioreceptors vary for SARS-CoV-2 wild type, mutant, trimeric and monomeric spike, and RBD. Further, Kd values may vary depending on the method and instrument used. So, this data should be interpreted carefully. **As most of the mutations have shown improved SARS-CoV-2 binding with ACE2. *** Due to antibody evasion of SARS-CoV-2. **** Mutation(s) in the epitope may affect specificity. However, detailed studies are needed.

## Data Availability

Manuscript does not generate any data. All sources are cited in the manuscript.
